# Feasibility, Acute Efficacy and Safety of Empirical Superior Vena Cava Isolation in Addition to Pulmonary Vein Isolation Using the Fourth-Generation Cryoballoon: Insights from a Randomized Trial

**DOI:** 10.3390/jcm14134422

**Published:** 2025-06-21

**Authors:** Vedran Pašara, Bruno Ban, Ivan Prepolec, Andrija Nekić, Zvonimir Katić, Domagoj Kardum, Davor Miličić, Vedran Velagić

**Affiliations:** 1 Department of Cardiovascular Diseases, University Hospital Centre Zagreb, 10000 Zagreb, Croatia; 2 School of Medicine, University of Zagreb, 10000 Zagreb, Croatia

**Keywords:** paroxysmal atrial fibrillation, pulmonary vein isolation, superior vena cava, fourth-generation cryoballoon, phrenic nerve injury, sinus node dysfunction

## Abstract

**Background/Objectives:** Pulmonary vein isolation (PVI) is the standard treatment for atrial fibrillation (AF), but medium-term success rates remain suboptimal. Non-pulmonary vein triggers, particularly from the superior vena cava (SVC), contribute to AF recurrence. Empirical SVC isolation (SVCi) in addition to standard PVI may improve outcomes. This study evaluated the acute procedural efficacy and safety of PVI with adjunctive SVCi versus PVI alone in patients with paroxysmal AF (PAF). **Methods:** In this randomized, controlled, single-center study, 149 patients with PAF were assigned to either standard PVI (*n* = 74) or PVI with adjunctive empirical SVCi (*n* = 75) using a fourth-generation CB. Primary endpoints were acute procedural success and the incidence of procedure-related complications, particularly phrenic nerve injury (PNI) and sinus node dysfunction. **Results:** Acute PVI was achieved in all patients; SVCi was successful in 84.9% of the PVI + SVCi group. Major complication rates were low and comparable between groups (0% vs. 2.6%, *p* = 0.157). However, the overall complication rate was significantly higher in the PVI + SVCi group (50.6% vs. 6.8%, *p* < 0.001), driven primarily by transient or impending right PNI (38.6% vs. 6.8%, *p* < 0.001) and sinus node dysfunction. All PNI events resolved before the end of the procedure. **Conclusions:** Empirical SVCi using a fourth-generation CB is feasible and generally safe, but carries a higher risk of transient PNI and reversible sinus node dysfunction. Therefore, CB SVCi should be approached with caution. Further studies are needed to evaluate long-term outcomes and assess whether the potential benefits outweigh these procedural risks.

## 1. Introduction

Pulmonary vein isolation (PVI) is the standard of care for patients with atrial fibrillation (AF) [1]. However, medium-term success rates remain suboptimal [2], prompting the exploration of novel treatment strategies—particularly those targeting ectopic foci outside the pulmonary veins. Growing evidence indicates that non-pulmonary vein (PV) triggers play a significant role in AF recurrence, emphasizing the need for adjunctive ablation strategies. The superior vena cava (SVC) is one of the most common non-PV origins of AF triggers, and several studies suggest that empirical superior vena cava isolation (SVCi), when performed in addition to PVI, may improve ablation outcomes in patients with paroxysmal atrial fibrillation (PAF) [3,4,5,6,7]. However, despite these promising findings, the benefit of additional SVC isolation remains debated, as randomized data are limited. While SVC ablation has been associated with potential complications such as phrenic nerve (PN) injury, sinus node dysfunction, and SVC stenosis, most studies report complication rates comparable to those observed with conventional cryoballoon (CB) PVI [6,7,8,9]. This study aimed to evaluate the safety profile and acute procedural efficacy of PVI followed by SVCi using a fourth-generation CB, compared to conventional CB PVI alone.

## 2. Materials and Methods

### 2.1. Study Design, Study Size, Patient Selection, and Randomization

This randomized, controlled, single-center cohort study included consecutive patients with PAF who were referred for CB ablation. The indication for invasive treatment of AF was determined independently of this study. Patients were prospectively enrolled and randomly assigned in a 1:1 ratio to either the experimental group, which underwent standard PVI with additional empirical SVCi, or the control group, which received standard PVI alone. The randomization sequence was generated using a computerized random number generator to ensure unbiased allocation. Due to the single-center nature of the study and the relatively homogeneous patient population, stratification by factors such as age, sex, or comorbidities was not performed. Allocation concealment was maintained by using sequentially numbered, sealed opaque envelopes, which were opened only after obtaining informed consent and immediately before the procedure. The operator was informed of the randomization outcomes, whereas patients were not notified of their assigned group in order to maintain blinding.

Exclusion criteria included age <18 years, cognitive or communication barriers preventing informed consent, previous AF ablation, significant left atrial dilation (diameter > 55 mm in the parasternal long-axis view), cardiomyopathy with manifest heart failure, unstable coronary artery disease, significant valvular heart disease, left atrial thrombus detected on preprocedural transesophageal echocardiography (TEE), severe renal failure, and other significant non-cardiac comorbidities.

The sample size was calculated to detect a medium effect size in the difference between two independent groups, with a significance level (α) of 0.05 and statistical power of 0.8. Using G*Power software (version 3.1.9.4), a minimum of 128 participants (64 per group) were required.

The study was approved by the Ethics Committee of the University Hospital Centre Zagreb and the Ethics Committee of the University of Zagreb School of Medicine. The protocol adhered to the ethical principles of the Declaration of Helsinki for medical research involving human subjects, ensuring participant privacy and data confidentiality. The study was registered at ClinicalTrials.gov (NCT05081310; 7 June 2021).

### 2.2. Preprocedural Assessment

A transthoracic echocardiogram was performed within three months prior to ablation to assess left ventricular ejection fraction and exclude significant structural heart or valvular disease. An uninterrupted anticoagulation strategy was implemented in all patients in accordance with guidelines [10]. Anticoagulation with vitamin K antagonists was maintained with a target international normalized ratio (INR) of 2.0–2.5, while direct oral anticoagulants (DOACs) were discontinued one day before the procedure and reintroduced at least four hours post-procedure.

### 2.3. Ablation Protocol

The PVI procedure was performed using a 28 mm CB (Arctic Front Advance Pro™ with Achieve Advance circular mapping catheter, Medtronic©, Minneapolis, MN, USA) as previously described [11]. Conscious sedation was achieved using a combination of fentanyl and midazolam.

Two venous access points were obtained via the right femoral vein. A 12 Fr sheath (FlexCath Advance, Medtronic Inc., Minneapolis, MN, USA) was introduced for the CB. A 10 Fr sheath was used for the intracardiac echocardiography (ICE) catheter (AcuNav Acuson, Siemens AG, Munich, Germany), which was utilized to visualize the left atrial appendage and assess potential anatomical variants of the PVs and was later exchanged for a decapolar electrophysiology catheter (Biotronik SE, Berlin, Germany) for PN pacing.

Transseptal access to the left atrium was obtained under fluoroscopic and ICE guidance using a BRK-1 needle (St. Jude Medical, Minneapolis, MN, USA) and an 8.5 Fr long sheath (Swartz, Abbott, Chicago, IL, USA). Unfractionated heparin (UHF) was administered immediately after transseptal puncture, targeting an activated clotting time (ACT) > 250 s, with repeated measurements and supplemental UHF boluses every 15–20 min.

PVI was performed in a standard fashion using a 28 mm fourth-generation CB (Arctic Front Advance Pro™, Medtronic©, Minneapolis, MN, USA) with a 20 mm octapolar intraluminal circular mapping catheter (Achieve Advance™, Medtronic©, Minneapolis, MN, USA), which was positioned at each pulmonary vein (PV) ostium to record baseline electrical activity. Using the “over-the-wire” technique, the CB was positioned at the PV antrum or ostium. PV occlusion was confirmed by contrast medium injection demonstrating complete retention without backflow into the left atrium. PVs were targeted in a clockwise fashion: left superior, left inferior, right inferior, and finally right superior. A single cryoenergy application lasting 3–4 min was delivered to each PV. When PV potentials were recorded, time to isolation (TTI) was noted. In cases of late (>60 s) or unsuccessful isolation, the CB was repositioned, and a second application was delivered. If real-time recordings were unavailable, a second 180 s freeze was applied, provided that the temperature of −40 °C had not been achieved within the first 60 s of the initial freeze [12]. When PV potentials were not visible during ablation to confirm isolation, the intraluminal circular mapping catheter was withdrawn to a more proximal position within the ostium, where electrical signals had been recorded prior to ablation [13]. In the presence of a common ostium, the standard approach was to sequentially address the superior and inferior branches, with one cryoapplication per branch.

PN function was monitored during right-sided PV ablation by ipsilateral PN pacing using a decapolar catheter placed in the right subclavian vein, with a 1500 ms cycle length and 20 mA output. Phrenic capture was confirmed by both tactile monitoring of diaphragmatic contractions (by placing the operator’s hand on the patient’s abdomen) and central venous pressure changes associated with diaphragmatic movement [14]. PN pacing was initiated once the CB temperature reached −20 °C to reduce the risk of balloon displacement caused by diaphragmatic movement. Cryoenergy application was immediately terminated in cases of impending PN paresis. A successful PVI was defined as the absence of or dissociation in PV potentials recorded with the intraluminal circular mapping catheter. Adenosine testing was not performed to assess dormant pulmonary vein conduction after ablation, as current guidelines do not recommend its routine use and its role is not addressed in the context of CB ablation [10].

**Figure 1:**
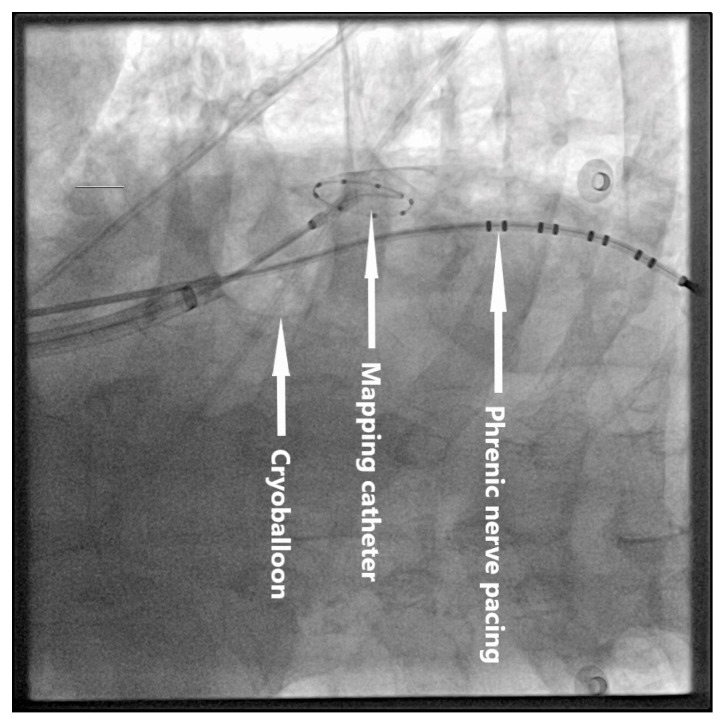
SVC ablation as seen in the posteroanterior view.

Following standard PVI, empirical isolation of the SVC was performed as described in previous studies [7,9,15]. The CB catheter was repositioned in the right atrium, and the circular mapping catheter was placed in the SVC. The CB was inflated in the right atrium and advanced toward the SVC ostium to achieve occlusion (Figure 1), which was confirmed by contrast injection. A single cryoapplication lasting 120–180 s was delivered. If SVC potentials were visible and TTI could be recorded, the cryoapplication was continued for 120 s after isolation was achieved. PN function was monitored during SVC ablation by ipsilateral PN pacing using a decapolar catheter placed in the right subclavian vein, with a 1200 ms cycle length—chosen due to increased risk of PN injury—and 20 mA output. Phrenic capture was confirmed by both tactile monitoring of diaphragmatic contractions (by placing the operator’s hand on the patient’s abdomen) and central venous pressure changes associated with diaphragmatic movement. This combined monitoring approach enabled early recognition of transient PNI, allowing prompt intervention to minimize the risk of permanent injury.

### 2.4. Post-Procedural Management

Standard post-procedural care was provided to all patients following PVI. Patients remained in the supine position until the following morning and underwent continuous telemetry monitoring until hospital discharge. All patients were discharged the day after the procedure. Prior to discharge, transthoracic echocardiography was performed to rule out any periprocedural complications.

### 2.5. Endpoints

The primary efficacy endpoints were the achievement of acute PVI and acute SVCi. The primary safety endpoint was the occurrence of any procedure-related complications, with particular focus on phrenic nerve injury and sinus node dysfunction.

### 2.6. Statistical Analysis

Categorical data are presented as absolute and relative frequencies. Differences in categorical variables were assessed using the Chi-square test. The normality of the distribution of numerical variables was evaluated with the Shapiro-Wilk test. Continuous variables were reported as median with interquartile range (IQR). Differences in continuous variables were analyzed using the Kruskal–Wallis test, followed by Conover’s post hoc test. All *p* values are two-tailed, and the level of statistical significance was set at alpha = 0.05. Statistical analyses were performed using MedCalc^®^ Statistical Software version 23.2.1 (MedCalc Software Ltd., Ostend, Belgium; https://www.medcalc.org; accessed on 22 January 2025).

## 3. Results

### 3.1. Study Population

A total of 149 patients were enrolled in the study between May 2021 and October 2023, of whom 93 (62.4%) were male. The median age was 62 years (IQR, 56–70), and the median CHA2DS2-VASc score was 2 (IQR, 1–3). All patients had PAF, with a median symptom duration of 18 months (IQR, 6–48). Patients were randomized to either PVI alone (*n* = 74) or PVI with additional SVCi (*n* = 75). Baseline characteristics are presented in Table 1.

### 3.2. Procedural Characteristics

The median procedure time was 54 min (IQR, 45–65), median fluoroscopy time was 6 min (IQR, 4–9), and the median total radiation dose was 9 mGy (IQR, 4–38). There were no statistically significant differences in procedural parameters between the two groups. The planned 180 s cryoapplication in the SVC was completed in 18 (24%) patients, while 48 (64%) patients received at least 120 s application. In most cases, the cryoapplication was prematurely interrupted due to signs of right PNI, sinus node dysfunction, or patient-reported pain. The median time to achieve SVCi was 22 s (IQR, 15–52.5), and the median temperature at isolation was −30.5 °C (IQR, −37.8–−18). The median temperature at 60 s was −43 °C (IQR, −48–−36), and the minimum temperature reached was −48 °C (IQR, −55–−41). Real-time recordings of SVC isolation were observed in 45 (60%) of patients. Additional procedural characteristics are summarized in Table 2.

### 3.3. Acute Procedural Efficacy and Safety Outcomes

Acute PVI was successfully achieved in all patients across both groups. SVCi was successfully achieved in 62 patients (84.9%) in the PVI + SVCi group. The most common reasons for premature termination of SVCi were impending right phrenic nerve injury (PNI) and loss of sinus rhythm. Notably, three patients in the PVI + SVCi group did not undergo SVC cryoablation due to PNI occurring during ablation of the right PVs. There was no statistically significant difference in the incidence of major complications between the two groups (0% vs. 2.6%; *p* = 0.157). However, two major complications were reported during the study period: one patient in the PVI + SVCi group required permanent AAI pacemaker implantation due to sinus node injury (SNI), and another experienced a major vascular complication involving both a pseudoaneurysm and an arteriovenous fistula, which required surgical repair.

The overall complication rate was 30.9% in the entire cohort and it was significantly higher in the PVI + SVCi group compared to the PVI-only group (50.6% vs. 6.8%; *p* < 0.001), driven primarily by a higher incidence of impending or transient right PNI. Impending or transient PNI occurred in 34 (22.8%) patients overall: 6.8% in the PVI-only group vs. 38.6% in the PVI + SVCi group (*p* < 0.001). Among affected patients in the PVI + SVCi group, 24 (82.8%) encountered impending or transient PNI during SVC ablation. In all cases, discontinuation of cryoenergy application led to complete recovery of phrenic nerve function before the end of the procedure. No cases of persistent PNI were observed. Bradycardia or junctional rhythm occurred during SVCi in 15 (20%) patients within the PVI + SVCi group; these events were not observed in the PVI-only group. Minor vascular complications (groin hematomas) occurred in two patients (1.3%). No pericardial complications or cerebrovascular events were reported.

## 4. Discussion

The main findings of the study are: (a) SVCi using a fourth-generation CB is feasible and can be successfully achieved in the majority of PAF patients; (b) the incidence of major complications is low and comparable between CB SVCi and conventional CB PVI; however, (c) CB SVCi is associated with a significantly higher rate of transient or impending PNI and reversible sinus node dysfunction compared to conventional CB PVI.

Over the past three decades, transcatheter PVI has become a widely accepted treatment strategy for AF, demonstrating superiority over antiarrhythmic drug therapy alone by significantly reducing arrhythmia recurrence and improving patients’ quality of life [16,17]. Although this approach has transformed AF management, mid- and long-term treatment outcomes remain suboptimal, with a substantial portion of patients experiencing AF recurrence. This has led to increasing interest in targeting non-PV triggers, which may be present in up to 28% of AF patients undergoing ablation [18,19].

Common non-PV trigger sites include the SVC, left atrial posterior free wall, crista terminalis, coronary sinus ostium, ligament of Marshall, and interatrial septum [18,20,21]. Kawai et al. reported that right atrial non-PV triggers are associated with PAF and disease progression, while left atrial non-PV triggers are more commonly associated with persistent or long-standing persistent AF [22]. SVC-related triggers have been identified in 6–12.9% of patients undergoing PAF ablation [4,6,20,23]. The arrhythmogenic potential of the SVC may be attributed to its shared embryologic origin with the sinus node and the presence of myocardial sleeves within the SVC which exhibit electrophysiological properties such as automaticity and triggered activity, both of which can initiate AF [24,25]. Accordingly, ablation strategies that implement adjunctive SVCi in addition to PVI may improve freedom from AF and enhance outcomes in PAF patients [5,7,22,26].

The SVC ablation carries specific safety risks and procedural challenges. Complications such as PNI and SNI are of particular concern due to the anatomical proximity of these structures to the SVC. In a histological study, Sánchez-Quintala et al. demonstrated that the right PN passes at an average distance of 0.3 ± 0.5 mm from the SVC [27]. Bohnen et al. further identified that the right PN capture most frequently occurs at a lateral, and less commonly at a posterolateral, portion of the SVC [28], supporting the rationale for an increased procedural risk of right PNI and SNI in this region.

Nonetheless, most studies utilizing thermal energy sources, either radiofrequency (RF) or cryoablation, for PVI with additional SVCi have reported relatively low overall complication rates, ranging from 4.5% to 8.8%, and a PNI incidence of up to 6%, with no significant difference between energy modalities [9,15,29,30,31]. These rates are comparable to the major complication rate of 4.7% reported for conventional CB PVI in a recent registry [32], as well as the 4.2% PNI rate reported in the YETI registry, which included over 17,000 patients [33]. Although SNI is rarely reported, Cheng et al. observed an SNI incidence of 4.5% following SVCi with RF, with one patient eventually requiring permanent pacemaker implantation [34].

In contrast, several studies employing CB ablation have reported higher complication rates, consistent with our findings. Wei et al. reported a transient PNI rate of 19.2% and an SNI rate of 7.7% using a second-generation CB [35]. Similarly, the recent CAVAC AF trial, which utilized a third-generation CB, reported a PNI rate of 13.3%, predominantly in patients who underwent combined PVI and SVCi, and an SNI rate of 18.8% [36]. In these studies, acute SVCi was successfully achieved in 80.8% and 93.8% of patients, respectively, which aligns with our results. Notably, the CAVAC AF trial reported a significantly longer fluoroscopy time in the PVI + SVCi group. In our cohort, however, procedure time, fluoroscopy time, and radiation dose remained comparable between the groups, despite the additional step of SVCi. Moreover, the overall fluoroscopy time was notably shorter than that reported in the CAVAC AF trial, likely due to the implementation of our previously described radiation reduction protocol [37]. This contrasts with other adjunctive ablation strategies, such as posterior left atrial wall isolation or left atrial appendage isolation, which typically prolong procedure times and increase radiation exposure due to additional mapping and lesion delivery [38,39]. These findings suggest that adjunctive SVCi may be implemented with minimal impact on workflow or resource utilization.

Evidence on the use of fourth-generation CB technology for SVC ablation is currently limited, and to the best of our knowledge, this is the first randomized controlled trial specifically evaluating its safety and feasibility for SVC ablation. Although the fourth-generation CB has demonstrated improved procedural efficiency and safety for PVI [40,41], its role in SVC ablation remains poorly established and associated with unique challenges.

While fourth-generation CB enables rapid and homogeneous tissue cooling, its enhanced performance may inadvertently increase the risk of adjacent tissue injury during SVC ablation. Although the incidence of major complications in our study was low, our findings confirm a higher rate of PNI and SNI, and do not support routine use of CB technology for SVC ablation. These findings highlight the need for risk mitigation strategies, including the use of three-dimensional electroanatomical mapping to accurately delineate the SVC-right atrium junction, activation mapping to identify the earliest site of sinus node activation, and procedural adjustments regarding energy source, energy application parameters, device selection, and patient selection [34,42,43].

Additionally, voltage mapping or delayed-enhancement imaging can provide valuable characterization of the atrial substrate, particularly atrial fibrosis—a key feature of atrial cardiomyopathy that influences AF pathophysiology, the relevance of non-PV triggers such as the SVC, and response to ablation. In this context, PVs may become electrically silent or less likely to serve as AF triggers due to scarring and loss of viable tissue, while non-PV triggers may assume a relatively greater role in initiating or sustaining AF [44,45]. Assessing fibrosis burden may help identify patients more likely to benefit from adjunctive ablation strategies like SVCi, thereby improving patient selection, procedural efficacy, and safety.

While the SVC is a recognized source of non-PV triggers, the rationale for empirical isolation remains debated. In our study, SVCi was performed empirically without prior demonstration of triggers, based on procedural feasibility and prior evidence suggesting potential benefit in PAF patients [5,6,7]. Currently, there are no well-established phenotypic or anatomical criteria to reliably identify patients who would benefit from ablation of non-PV triggers. In the absence of rhythm outcome data, any clinical advantage of empirical SVCi in our cohort remains speculative. Until long-term follow-up results are available, the decision to perform empirical SVCi—particularly in light of increased complication rates—should be approached with caution.

Follow-up data from our cohort may help clarify whether the PVI + SVCi approach provides a significant arrhythmia-free survival benefit over PVI alone that could potentially outweigh the associated procedural risks. In the absence of robust long-term rhythm outcome data, the risk-benefit balance of empirical CB SVCi remains uncertain in PAF patients. However, emerging evidence suggests that pulsed-field ablation (PFA), particularly when guided by intracardiac echocardiography, offers effective SVCi with a favorable safety profile and minimal risk of collateral injury [46]. Future studies directly comparing ICE-guided PFA with cryothermal energy for SVCi are warranted to better define the optimal approach in this patient population.

## 5. Conclusions

This randomized study demonstrates that SVCi using a fourth-generation CB is feasible and can be successfully achieved in the majority of patients with PAF. While the incidence of major complications was low and comparable between groups, the addition of CB SVCi to PVI was associated with a significantly higher rate of transient or impending right phrenic nerve injury and reversible sinus node dysfunction. These findings suggest that the routine application of CB-based SVCi should be approached with caution, and underscore the need for further refinement of procedural techniques and risk mitigation strategies to improve safety. Future studies are needed to determine whether the potential benefits of adjunctive SVCi in terms of long-term arrhythmia control justify the associated procedural risks.

## 6. Limitations

This study has several limitations. First, it was conducted at a single center with a relatively limited sample size, which may affect the generalizability of the findings. Second, although procedures were performed by experienced operators, the results may not be directly applicable to centers with less familiarity with fourth-generation CB technology. Third, the study did not incorporate direct assessment of atrial fibrosis, such as voltage mapping or delayed-enhancement imaging, which limits the ability to determine whether the observed efficacy and safety profiles were influenced by differences in atrial substrate among patients. Additionally, there are currently no well-established phenotypic or anatomical criteria to guide selection of patients who might benefit most from empirical non-PV trigger ablation strategies, including SVCi. Fourth, this study focused on feasibility, acute efficacy, and procedural safety; therefore, long-term outcomes such as arrhythmia recurrence or durable lesion formation were not assessed here and will be reported in a separate follow-up study. Finally, the inclusion of only patients with paroxysmal atrial fibrillation may limit the extrapolation of results to other AF populations, such as those with persistent or long-standing persistent AF.

## Figures and Tables

**Table 1:** Baseline characteristics.

	All (*n* = 149)	PVI-Only (*n* = 74)	PVI + SVCi (*n* = 75)	Difference (95% CI)	*p* Value *
Age, y	63 (56–70)	63 (55–70)	63 (58–70)	1 (−3–4)	0.66
Male, *n* (%)	93 (62.4)	45 (60.8)	48 (64)	*n*/A	0.69 ^†^
BMI, kg/m^2^	27.9 (24.8–31.2)	27.6 (24.2–30.4)	29.3 (24.5–31.8)	1.1 (−0.4–2.5)	0.15
CHA2DS2-VASc score	2 (1–3)	2 (1–3)	2 (1–3)	0 (0–1)	0.52
AF history, mo	18 (6–48)	18 (6–48)	16 (6–45)	−1 (−7–3)	0.57
Arterial hypertension, *n* (%)	122 (81.9)	59 (79.7)	63 (84)	N/A	0.50 ^†^
Diabetes mellitus, *n* (%)	24 (16.1)	10 (13.5)	14 (18.7)	N/A	0.39 ^†^
Dyslipidemia, *n* (%)	86 (57.7)	41 (55.4)	45 (60)	N/A	0.57 ^†^
CKD, *n* (%)	10 (6.7)	4 (5.4)	6 (8)	N/A	0.53 ^†^
COPD, *n* (%)	4 (2.7)	1 (1.4)	3 (4)	N/A	0.32 ^†^
OSAS, *n* (%)	4 (2.7)	1 (1.4)	3 (4)	N/A	0.32 ^†^
CAD, *n* (%)	11 (7.4)	3 (4.1)	8 (10.7)	N/A	0.12 ^†^
TIA/CVA, *n* (%)	6 (4)	2 (2.7)	4 (5.3)	N/A	0.68 ^†^
Smoking, *n* (%)	32 (21.5)	14 (18.9)	18 (24)	N/A	0.45 ^†^
LVEF, %	60 (60–65)	60 (60–65)	60 (60–65)	0 (0–0)	0.54
LAD, mm	39 (33.8–43.3)	39 (34–43.2)	41.5 (36–44.5)	2 (−1–5)	0.19
LAVI, mL/m^2^	32.5 (26–40.4)	32 (27–38)	33.8 (25.9–40.0)	1 (−3.3–5.5)	0.58

* Kruskal–Wallis test; ^†^ Chi-square test; Differences between groups with 95% CIs are reported for continuous variables only; For non-continuous variables, “N/A” indicates that a difference and 95% CI are not applicable; AF, atrial fibrillation; BMI, body mass index; CI, confidence interval; CKD, chronic kidney disease; COPD, chronic obstructive pulmonary disease; CVA, cardiovascular accident; LAD, left atrium diameter; LAVI, left atrium volume index; LVEF, left ventricular ejection fraction; N/A, not applicable; TIA, transient ischemic attack.

**Table 2:** Procedural characteristics.

	All (*n* = 149)	PVI-Only (*n* = 74)	PVI + SVCi (*n* = 75)	95% CI (PVI + SCVi)	Difference (95% CI)	*p* Value *
Procedure time, min	54 (45–65)	55 (44–65)	53 (45–65)	-	0 (−5–5)	0.70
Fluoroscopy time, min	6 (4–9)	6 (3.9–9.6)	5 (4.0–8.0)	-	−0.5 (−1.5–0.5)	0.45
Total radiation dose, mGy	9 (4–38)	12 (4–51.2)	6 (3.8–22)	-	−2 (−7–1)	0.29
SVC freezing time, s	-	-	131.5 (92–179)	-		N/A
Time to SVC isolation, s	-	-	22 (15–52.5)	28.3–53.1	-	N/A
Temperature at SVC isolation, °C	-	-	−30.5 (−37.8–−18)	−32.5–−23.3	-	N/A
Nadir SVC temperature, °C	-	-	−48 (−55–−41)	−51.3–−47.1	-	N/A

* Kruskal–Wallis test; Differences between groups with 95% CIs are reported for parameters measured in both groups; For SVC-specific parameters, which are applicable only to the PVI + SVCi group, within-group 95% CIs are presented; “N/A” indicates not applicable for parameters not measured in a group. CI, confidence interval; N/A, not applicable; PVI, pulmonary vein isolation; SVC, superior vena cava; SVCi, superior vena cava isolation.

## Data Availability

Data supporting the findings of this study are available from the corresponding author upon reasonable request.

## Abbreviations

The following abbreviations are used in this manuscript:

ACT	activated clotting time
AF	atrial fibrillation
CB	cryoballoon
DOAC	direct oral anticoagulant
ICE	intracardiac echocardiography
INR	international normalized ratio
IQR	interquartile range
LA	left atrium
PAF	paroxysmal atrial fibrillation
PFA	pulsed-field ablation
PN	phrenic nerve
PNI	phrenic nerve injury
PVI	pulmonary vein isolation
PV	pulmonary vein
SNI	sinus node injury
SVC	superior vena cava
SVCi	superior vena cava isolation
TEE	transesophageal echocardiography
TTI	time to isolation
UHF	unfractionated heparin
